# Highly Filled Flowable Composite Resins as Sole Restorative Materials: A Systematic Review

**DOI:** 10.3390/ma18143370

**Published:** 2025-07-18

**Authors:** Konstantinos Tzimas, Eftychia Pappa, Maria Fostiropoulou, Efstratios Papazoglou, Christos Rahiotis

**Affiliations:** Department of Operative Dentistry, National and Kapodistrian University of Athens, 11527 Athens, Greece; kwstastzimas@dent.uoa.gr (K.T.); effiepappa@dent.uoa.gr (E.P.); mariafos@dent.uoa.gr (M.F.); spapazog@dent.uoa.gr (E.P.)

**Keywords:** flowable composite, dental material, operative dentistry, systematic review, biomaterials

## Abstract

The continuous need for simplified, minimally invasive restorative procedures with a high precision has led to the advancement of highly filled flowable resin-based materials. These materials present excellent initial outcomes in various clinical applications, including the injection molding technique. Given that several clinical reports present signs of wear and staining, this systematic review aims to investigate the mechanical and optical properties of highly filled flowable composite resins. A comprehensive literature research was conducted to identify relevant studies from the PubMed, the Cochrane Library, and Scopus databases. Data extraction and screening was performed by two independent evaluators. Both in vitro studies and clinical trials were included. A total of thirty-one studies were included in this review. A total of 27 in vitro studies investigated highly filled flowable composite resins independently, or in comparison with conventional composite resins, traditional flowable composites, bulk-fill flowable composites, glass ionomer cements, and compomers. Additionally, four randomized controlled clinical trials (RCTs) compared highly filled flowable composite resins with their conventional counterparts. Highly filled flowable composite resins exhibit adequate optical properties. Despite their significant improvements, their mechanical properties remain inferior to those of medium-viscosity composite resins. These materials demonstrate a favorable initial performance in the injection molding technique. Based on a limited number of RCTs, these materials demonstrate an adequate performance in class I and II restorations; however these findings should be interpreted with caution. The reported drawbacks in laboratory studies may contraindicate their clinical application in extensive cavities, load-bearing areas, and in cases of excessive tooth wear and parafunctional activity. A careful clinical case selection is strongly recommended.

## 1. Introduction

Conventional, medium-viscosity composite resins are the most commonly used materials for both anterior and posterior restorations [1,2]. Conservation of tooth structure, an adequate color adaptability, satisfactory mechanical properties, and the lower cost compared to indirect restorative counterparts are listed as the benefits of these biomaterials [3,4]. However, their inferior rheological characteristics result in marginal defects, due to a poor adaptation to cavity walls and voids between increments [5]. To confine these drawbacks, first-generation flowable composites were launched in the 1990s, which contained 20–25% less filler loading compared to medium-viscosity composite resins [6,7]. Their low viscosity was conducive to ease of use, improved handling and wetting properties, a good adaptability to cavity walls, and restricted air bubble entrapment in their mass [8,9]. On the other hand, their mechanical and optical properties were impaired by their reduced filler content [10], limiting their use to only as class V restorative materials, liners, pit and fissure sealants, and as materials for marginal restoration repair [6]. Their high volumetric polymerization shrinkage rate of approximately 5% proved to be an additional disadvantage of traditional flowable composite resins [5].

In response to clinicians’ demands for simplicity and the durability of final restorations, dental material manufacturers developed novel flowable composites, suitable for a wide range of clinical cases. Their indications for use include the treatment of tooth wear and tooth erosion, correction of tooth shape and size, smile rejuvenation, and cuspal restoration, without the need for capping layers [11,12,13,14]. Their advanced formulation includes a higher filler loading, optimum filler size, the incorporation of refined monomers, and the pre-treatment of filler particles. Manufacturers suggest that these modifications enhance the materials’ physical and mechanical properties [11]. Simultaneously, superior marginal adaptation is maintained [15]. Their higher filler content—ranging from approximately 61% to 71% by weight—enables them to withstand high occlusal loads [16,17]. Until recently, these statements have not been completely verified. In the literature, this type of flowable composite resin is primarily reported as highly filled flowable composite resin [18,19,20]. The terms “next-generation composites” [21] and “injectable composites” [22] are often used interchangeably, which can lead to confusion.

The use of highly filled flowable composite resins as stand-alone restorative materials—particularly in the context of the injection molding technique—has gained significant attention in recent years [23,24,25,26]. Introduced by Terry and Powers in 2014, the injection molding technique is described as a novel restorative technique that transforms an analog or digital wax-up into a final composite resin restoration, with minimal or no tooth preparation [23,24]. One or more transparent silicone indices based on a wax-up enable the passive insertion of the restorative material, minimizing the risk of restoration distortion. The accurate reproduction of anatomical contours is reported as the main advantage of this technique [27]. Nowadays, the injection molding technique, utilizing highly filled flowable composite resins, is used in a wide range of clinical cases, including single-tooth restorations, tooth recontouring, the replacement of missing teeth with direct composite resin-bonded fixed dental prostheses, and full-mouth rehabilitation—with or without alteration of the vertical dimension [23,24,28,29,30,31,32,33,34,35,36,37,38,39,40,41,42,43,44,45,46,47,48,49,50]. When minimal tooth structure loss is evident, highly filled flowable composite resins have performed optimally [24,34,36,44]. However, several clinical cases reported signs of staining on the material’s surface and/or at the tooth–material interface, minor chippings, and surface voids [30,43]. These observations highlight the inferior performance of highly filled flowable composite resins compared to other commonly used restorative materials.

This systematic review aims to address the following question: Do highly filled flowable composite resins exhibit adequate mechanical and optical properties and a good clinical performance? The use of these materials in novel restorative techniques and their promotion by manufacturers as sole restorative materials highlight the need to understand both their potential and limitations. This knowledge is crucial for appropriate case selection, thereby ensuring the long-term durability of these materials.

## 2. Materials and Methods

**Figure 1:**
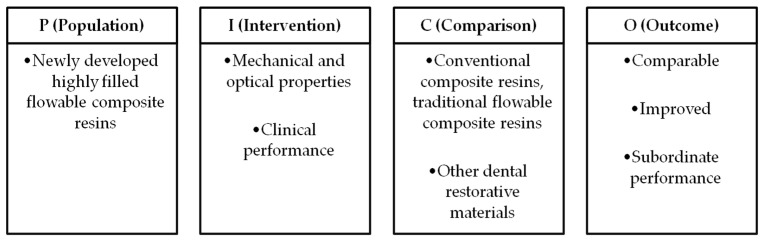
PICO framework of systematic reviews.

A systematic review was conducted to assess the mechanical properties, optical characteristics, and clinical performance of highly filled flowable composite resins. This report followed the guidelines of the latest Preferred Reporting Items for Systematic Reviews and Meta-Analyses statement (PRISMA) [51]. The present systematic review was registered on the PROSPERO platform (ID number: 1063216). The PICO framework was adapted to formulate the research question and facilitate the selection process (Figure 1).

### 2.1. Sources of Information and Search Strategy

A systematic literature search was conducted using the following electronic databases: MEDLINE via PubMed, Scopus, and the Cochrane Library. A manual search of reference listings has been applied. The search covered publications from the databases’ inception through to February 2025. The synthesis of information from diverse sources, including qualitative and quantitative studies, has been performed. The search strategy is presented in Table 1.

### 2.2. Eligibility Criteria

The search results were filtered by language, and studies in English were eligible. This review included randomized controlled clinical trials and in vitro studies. Laboratory studies that incorporated at least one highly filled flowable composite resin in their methodology were included. Clinical trials in progress and clinical trials not using conventional composite resins as controls were excluded. Studies that exclusively evaluated polymerization shrinkage, depth of cure, and the degree of conversion of highly filled flowable composite resins—without simultaneously evaluating mechanical and optical properties—were excluded at the stage of full-text screening. This review also excluded case reports, case series, review articles, short communications, patents, and conference proceedings.

### 2.3. Data Extraction, Screening, and Charting

Screening was conducted by two independent reviewers (K.T. and C.R.) in two phases—first by title and abstract and then by full text—based on the pre-established eligibility criteria. In the presence of a discrepancy, screening was conducted by a third reviewer (E.P.) until consensus was reached. Automated screening using AI-powered tools was performed for studies originating from the PubMed and Scopus databases [52]. Studies retrieved from the Cochrane Library database were screened manually. Short communications, conference proceedings, and clinical trials in progress were excluded after manual screening. The reference lists of all the included articles were manually searched to identify further eligible studies.

Data charting was conducted by two examiners. The first examiner (K.T.) extracted the relevant data from the eligible studies, while the second examiner (C.R.) verified its accuracy. The results are organized chronologically in tables. Tables for in vitro studies include the author and year of publication, dental materials used, tested parameters, and key findings. Tables for randomized controlled clinical trials present the author and year of publication, objectives, materials used, sample size, follow-up intervals, evaluation criteria, and observed outcomes.

### 2.4. Risk of Bias Assessment

The Johanna Briggs Institute (JBI) critical appraisal tools were used to assess the trustworthiness and relevance of the included studies. More precisely, the JBI Critical Appraisal Checklist for Randomized Controlled Clinical Trials (RCTs) was used to evaluate the RCTs [53], and the JBI Checklist for Quasi-Experimental Studies was used for the evaluation of the included in vitro studies [54]. The JBI SUMARI (System for the Unified Management, Assessment, and Review of Information) (https://sumari.jbi.global/, accessed on 25 March 2025) software platform was used to export summary tables following the completion of the critical appraisal. Two independent reviewers (K.T. and C.R.) conducted the assessments of the eligible studies. Discrepancies were resolved through discussion, until a consensus was reached, thereby enhancing the reliability and validity of the systematic review’s key findings.

## 3. Results

**Figure 2:**
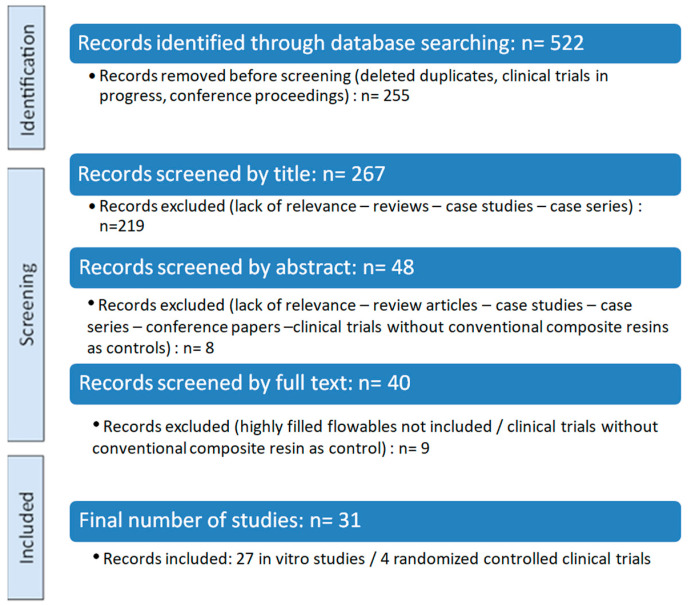
PRISMA flow diagram.

A flow chart illustrating the number of studies identified and screened is shown in Figure 2.

Out of 522 records identified, 31 studies were included in this systematic review after full-text screening. The studies were divided into in vitro studies and randomized controlled clinical trials, followed by a synthesis of their key finding. A summary of the eligible studies, including their research type and investigated outcomes, is presented in Table 2.

### 3.1. In Vitro Studies on Highly Filled Flowable Composite Resins

This review included 27 in vitro studies [17,55,56,57,58,59,60,61,62,63,64,65,66,67,68,69,70,71,72,73,74,75,76,77,78,79,80]. Each study evaluated one or multiple material characteristics. The in vitro studies were subcategorized into two groups: the first group included 15 in vitro studies that incorporated conventional composite resins in their methodology [17,55,56,57,58,59,60,61,62,63,64,65,66,67,68], and the second group consisted of 12 in vitro studies that examined either a single highly filled flowable composite resin or highly filled flowable composite resins alongside traditional flowable composites, bulk-fill flowable composites, glass ionomer cements, and compomers [69,70,71,72,73,74,75,76,77,78,79,80]. Detailed descriptions of the studies’ characteristics and qualitative findings are presented in Table 3 and Table 4.

#### 3.1.1. Optical Properties and Color Stability of Highly Filled Flowable Composite Resins

The findings on optical properties indicate that highly filled flowable composite resins exhibit an acceptable color stability, supporting their potential use in esthetically demanding restorations. Initially, Nair et al. demonstrated that a highly filled flowable composite resin performed inferiorly compared to nanofilled and nanohybrid conventional composites [55]. Owing to recent developments in highly filled flowable composites, no statistically significant differences were found in color stability among highly filled flowable composite resins, a bulk-fill flowable composite, and a conventional nanofilled composite resin [56]. Interestingly, Tüter Bayrakrat et al. reported that conventional composite resins had lower fluorescence (ΔE_FI_) and color adjustment (ΔE_CP_) levels compared to highly filled flowables in class V cavities [62]. Color changes due to toothbrushing abrasion remained within acceptable thresholds when highly filled flowables were compared with traditional and self-adhesive flowables [69].

#### 3.1.2. Surface Characteristics of Highly Filled Flowable Composite Resins

Highly filled flowable composite resins exhibited statistically comparable surface roughness values (arithmetic average of the absolute values of the surface height deviations measured from the mean line, Ra) to those of conventional nanohybrid composites [60,64,65]. In a study by Elsahn et al. in 2023, G-aenial Universal Injectable exhibited lower Ra values, even when compared to a CAD/CAM resin-based material, when used as thin occlusal veneers [74]. Vulović et al. demonstrated that highly filled flowable composite resins, consisting of ultra-fine barium or strontium fillers combined with full-coverage silane technology (G-aenial Universal Injectable, GC, and G-aenial Universal Flo, GC) exhibited lower surface roughness values compared to other highly filled flowables [76,80]. Surface roughness scores were affected by both polishing systems and the acidic challenges implemented in the in vitro methodological protocols of the studies [75,76,80]. Regarding surface gloss, G-aenial Universal Injectable performed favorably compared to other traditional flowables available on the market [69].

#### 3.1.3. Mechanical Characteristics of Highly Filled Flowable Composite Resins

While notable improvements in microhardness, flexural strength, and wear resistance have been reported for highly filled flowable composite resins, divergent results across studies raise concerns regarding their performance. Multiple in vitro studies reported that highly filled flowable restorative materials exhibited an inferior Vickers microhardness compared to conventional microfilled composites, nanofilled composites, bulk-fill composite resins, and CAD/CAM resin-based materials [55,57,59,65,74]. Conversely, when compared with traditional flowable composites, highly filled flowable restorative materials exhibited superior hardness values [73]. Among several highly filled flowable resin composites, G-aenial Universal Injectable presented the highest surface hardness values [80]. Controversial findings were observed regarding the elastic modulus of highly filled flowable composite resins. Imai et al. demonstrated lower elastic modulus values for highly filled flowable composite resins compared to microhybrid and nanofilled conventional composite resins [17], while Degirmenci et al. and Rajabi et al. showed higher flexural strength and elastic modulus values for highly filled flowable materials compared to microhybrid and nanohybrid conventional composites [57,66]. In a recent study, no differences in flexural strength values were observed between highly filled flowables and a nanohybrid conventional composite resin [65]. Highly filled flowable composites outperformed traditional flowables and bulk-fill flowable composites in terms of flexural strength and elastic modulus [72].

Regarding wear resistance, controversial findings have once again been reported. Turk et al. demonstrated that conventional nanohybrid and nanofilled composite resins outperformed flowable composite resins in terms of wear volume loss and maximum wear depths [61]. However, G-aenial Universal Injectable by GC showed a lower wear volume loss than that of a CAD/CAM milled resin-based material when used as 1 mm thin occlusal veneers [74]. Recent data reveal a significant similarity of wear patters between highly filled flowable composites and conventional composite resins. Therefore, several researchers have supported the use of highly filled flowable composite resins in occlusal, load-bearing areas [64,66].

### 3.2. Randomized Controlled Clinical Trials on Highly Filled Flowable Composite Resins

To date, only four randomized controlled clinical trials have assessed the clinical performance of highly filled flowable resin-based materials compared to conventional composite resins [81,82,83,84]. Three studies focused on the clinical performance of highly filled flowable resin composites in class I and II restorations of varying sizes [81,83,84], while one investigated non-cavitated cervical lesions [82]. Regarding function and esthetics, both materials performed equally well. Concerning marginal adaptation and surface gloss, highly filled flowable composite resins have shown superior performance relative to controls [82,84]. The clinical trials are summarized in Table 5.

### 3.3. Risk of Bias of Included Studies

**Figure 3:**
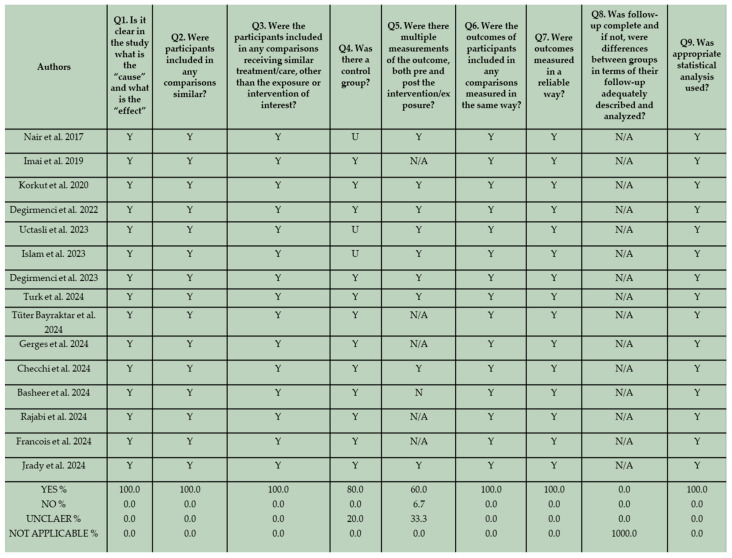
JBI Critical Appraisal results for the first group of in vitro studies that incorporated conventional composite resins in their methodological framework [17,55,56,57,58,59,60,61,62,63,64,65,66,67,68].

**Figure 4:**
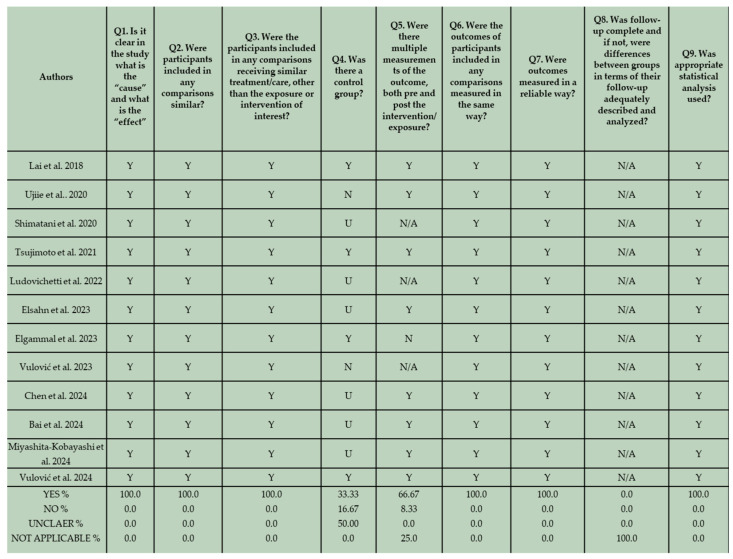
JBI Critical Appraisal results for the second group of in vitro studies that do not employ conventional composite resins in their methodological framework [69,70,71,72,73,74,75,76,77,78,79,80].

**Figure 5:**
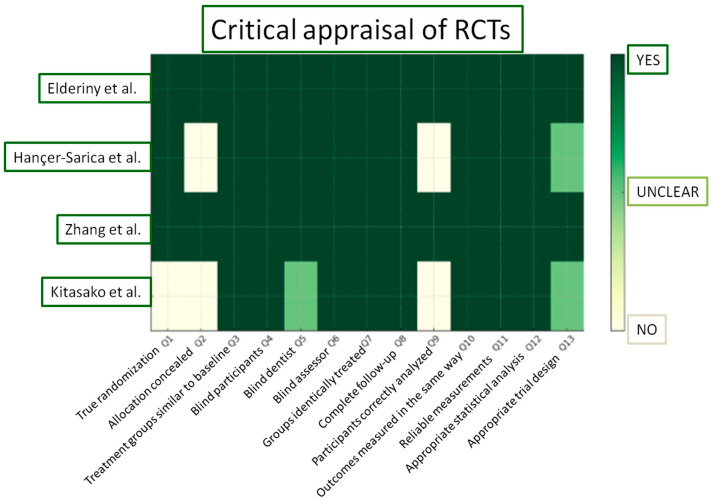
JBI Critical Appraisal results for the included RCTs [81,82,83,84].

The risk of bias of the in vitro studies and randomized controlled clinical trials was assessed by two reviewers (K.T. and C.R) independently, based on a fixed categorization relative to the design. The two groups of in vitro studies were evaluated separately. The results are shown in Figure 3, Figure 4, and Figure 5 respectively.

In terms of reporting quality, no bias related to temporal precedence was identified, since all the studies clearly presented “cause” and “effect” variables. Furthermore, there was no indication of bias related to confounding factors and the administration of interventions. This is based on the fact that in all the in vitro studies, specimens were identically prepared and received similar care, aside from the exposure to the intervention. Regarding the potential of risk of bias related to selection and allocation, 80% of the eligible studies that incorporate conventional composite resins in their methodological design, and 33.33% of the included studies that did not employ conventional composite resins in their methodology, provided well-defined control groups. In several studies, an unclear assessment of control groups is evident. Although control groups could be identified based on the formulated null hypothesis, they were not adequately described in the manuscripts. Additionally, not all eligible in vitro studies incorporated both pre- and post-intervention measurements of the examined outcome in their methodological design. This may be attributed to the nature of the design and should not be interpreted as indicative of a high risk of bias. For instance, wear and volume loss can only be assessed after wear simulation. In two studies no pre-intervention measurements were conducted. In these studies surface roughness, hardness, and gloss were assessed after thermocycling and after immersion in different beverages [65,75]. Nevertheless, due to the implementation of standardized and reliable measurement techniques, the risk of bias related to the assessment, detection, and measurement of the outcome appeared low. A bias related to participant retention is not applicable in this context, as all laboratory specimens were accessible for assessment. Lastly, the validity of the applied statistical analysis proved to be high for all the in vitro studies, demonstrating adherence to well-defined analytical standards.

In this section, according to the JBI Checklist for Randomized Controlled Clinical Trials, the studies were evaluated using the scoring criteria “yes”, “no”, “unclear”, “not applicable”, based on the data extracted after the study evaluation. Enlarging on the risk of bias, the study of Kitasako et al. displayed the most significant drawbacks [81]. Although dice were used for randomization, assessors proceeded to modifications of the randomization process when more than one restoration was required for a single patient. Furthermore, no information on allocation concealment was provided in two out of four clinical trials [81,84]. In the study of Kitasako et al. [81], even though participants unavailable for further evaluation were well-defined, they were censored from the total count of restorations initially inserted and consequently from the statistical analysis. Two clinical trials demonstrated an overall low risk of bias [82,83].

## 4. Discussion

The results of this review should be interpreted with caution. Variations were observed among the studies examining one highly filled flowable material, as well as across studies evaluating different highly filled flowable composite resins or different restorative materials (e.g., highly filled flowable materials, traditional flowable materials, bulk-fill materials, compomers, glass ionomer cements, and conventional composite resins). These discrepancies present a multifactorial and interdependent etiological pattern.

### 4.1. Factors Influencing Optical and Mechanical Properties of Dental Biomaterials

The mechanical, physical, optical, and surface characteristics of dental materials are predominantly related to their unique composition. The structure of the organic matrix, the composition of inorganic filler particles, the filler-to-resin matrix ratio, and the silanization of the filler components are key factors that affect their properties. The material’s composition significantly affects its further properties, such as the degree of conversion, water sorption, and solubility [17,59,85,86]. Beyond the material’s composition, finishing and polishing procedures, methodological variations, sample fabrication techniques, storage and testing conditions, and external aggravating stimuli (colorant solutions, acidic and abrasive challenges) can significantly affect the laboratory performance of dental materials [56,57,58,60,66,75,76,80].

#### 4.1.1. The Predominant Effect of Inorganic Filler Content on Optical Properties and Microhardness

The optical performance of composite resins is influenced by factors such as surface roughness, gloss, and hardness [87]. Furthermore, surface roughness is affected by filler content, filler type and size, the surface area occupied by filler particles, the degree of conversion, filler–matrix interactions, the silane coupling agents, and ultimately, the material’s hardness [88,89,90]. While colorant solutions and abrasive challenges negatively affect the microhardness and color stability of highly filled flowable resin composites [55], variances in microhardness values may be attributed to the type and distribution of fillers. For instance, the strontium glass fillers found in G-aenial Universal Flo are associated with inferior physical properties and a more complex attachment to the organic matrix than the zirconia and silica fillers incorporated in conventional composite resins [55,91]. A uniform filler distribution contributes to the enhancement of physical and optical properties [59]. The filler size in G-aenial Universal Flo by GC (highly filled flowable) ranges from 16 to 200 nm, whereas Filtek Z350XT (conventional composite resin) contains filler particles ranging from 4 to 20 nm in size [92,93]. These variations may explain the differences in the mechanical properties between these materials.

The optical performance of composite resins is related to translucency, opalescence, chroma, and the refractive indices of both monomers and fillers [94]. Differences in filler type and inorganic composition can significantly affect translucency [95,96]. This fact is endorsed by Bai et al. in 2024, who demonstrated that G-aenial Universal Injectable (GC) exhibited a higher translucency when compared to Filtek Supreme Flowable (3M ESPE), likely due to the imperfect refractive index of its zirconia fillers [78]. G-aenial Universal Injectable, filled with barium glass, displayed the highest translucency and opalescence values when compared with G-aenial Universal Flo, which is filled with strontium glass, and G-aenial Anterior, a microhybrid conventional resin composite, filled with strontium glass and lanthanoid fluoride [60]. Furthermore, the abrasive hardness of polishing systems is directly related to translucency values [97]. Diamond particles, presenting a higher abrasive hardness than aluminum oxide particles, result in an extended abrasion of the organic matrix and increased filler protrusion. This fact partially explains the variations in translucency even within the same material [98,99]. It should be noted that while a higher filler content improves strength and wear resistance, it ultimately reduces translucency and increases opacity [100,101,102,103].

#### 4.1.2. Influence of Inorganic Filler Content on Surface Characteristics

The filler’s loading, shape, and size could partially explain variations in surface roughness and gloss values among composite resins [90,104]. Lower filler loading, an irregular filler shape, a heterogeneous filler composition, and larger average particle sizes are inextricably linked to an increased surface roughness and decreased surface gloss. This is validated by the findings of Miyashita–Kobayashi et al., who compared the surface roughness and gloss of two highly filled flowable composite resins and found differences among them, with a material consisting of supra-nanospherical fillers and an average particle size of 0.2 μm exhibiting a superior surface gloss and smoother surfaces compared to a material containing irregularly shaped fillers and an average particle size of 0.8 μm [79]. Beautifil Injectable X consists of bioactive surface pre-reacted glass ionomer (S-PRG) fillers with an average particle size of 0.8 μm. Conversely, G-aenial Universal Flo and G-aenial Universal Injectable consist of strontium and barium glass fillers, respectively, along with silica particles, forming homogeneous filler patterns. In contrast, Tetric EvoFlow and Filtek Supreme Flowable Restorative incorporate Ytterbium trifluoride (YBF_3_) and a mixture of three to four diverse filler types, leading to a heterogeneous composition that negatively impacts surface roughness values [76]. At this point, it is essential to critically assess that most in vitro studies analyze the surface roughness using a single height parameter (Ra value). However, the inclusion of spatial, functional, or hybrid (e.g., developed interfacial area ratio, Sdr) parameters can offer a greater insight into the surface texture of previously analyzed highly filled flowable composite resins [105].

#### 4.1.3. The Interaction Between Inorganic Filler Content, Organic Matrix Composition, and the Oral Environment in the Mechanical Performance of Dental Biomaterials

Mechanical properties—particularly wear resistance—depend on the filler type, shape, and size; interfiller spacing; and the type of filler pre-treatment [85,106]. Additionally, characteristics such as the degree of conversion, resistance to hydrolytic degradation, water sorption, and finishing and polishing procedures play a pivotal role in wear resistance and flexural strength [6,10,15,17,70,107,108,109]. The smaller particle size of several highly filled flowables results in lower friction coefficients and restricted internal shear stresses at the polymer matrix, potentially explaining the favorable wear resistance among several highly filled flowable composite resins and bulk-fill flowables [15,70,110]. Moreover, the small interparticle spacing among small-sized fillers may form a protective barrier to the resin matrix. The lower volumetric wear of G-aenial Universal Injectable compared to a resin-based CAD/CAM material in 1 mm thin veneers should be interpreted with caution. Manufacturers recommend a minimum thickness of 1.5 mm for this CAD/CAM material [111], highlighting notable limitations in the experimental design of the included study. The increased filler content and the high concentration of Bis-GMA monomers in conventional composite resins improve their mechanical and physical properties [112,113], thereby justifying the superior wear performance of conventional composite resins compared to highly filled flowable composite resins. The finding that G-aenial Universal Flo (GC) demonstrated significantly comparable flexural strength values to those of a nanofilled composite resin suggests that filler loading alone does not enhance mechanical strength. Different monomers have unique molecular weights and viscosities, directly influencing the material’s mechanical behavior [114].

Upon exposure to oral conditions, dimensional changes, degradation, and weakening of the bond between organic and inorganic components occur [17,115]. A higher filler content is related to reduced water sorption, due to the increased hydrophobicity of the material [116,117]. However, a higher filler content may lead to increased water sorption, due to the greater surface area available for water uptake. Different types of monomers and their quantitative allocation in the organic matrix influence the water sorption behavior of biomaterials [118,119]. A higher Bis-GMA and TEGDMA content provides greater susceptibility to hydrolytic degradation compared to UDMA monomers [120,121]. TEGDMA absorbs more water than Bis-GMA due to its greater flexibility and intermolecular spacing [122,123,124]. The water sorption promotes the hydrolysis of the coupling agent, weakening the filler–matrix bond [125]. By leveling up the amount of nano-sized fillers and creating a homogeneous distribution of inorganic particles, the internal volume available for water infiltration is reduced. This fact partially explains the lower water sorption values of various conventional composite resins compared to highly filled flowables. Nevertheless, disparities are present among different highly filled flowables, primarily due to variations in their organic and inorganic composition. For example, Beautifil Injectable X by Shofu exhibits higher water sorption and degradation levels than G–Aenial Universal Injectable under acidic conditions. This can be attributed to the higher quantity of Bis–GMA and TEGDMA in its organic matrix and the presence of fluorosilicate glass fillers (S-PRG fillers) [14]. This filler type exhibits a higher susceptibility to degradation by weak acids [88,126].

### 4.2. Limitations of the In Vitro Studies and Randomized Controlled Clinical Trials

Systematic reviews provide an essential input into clinically relevant queries by compiling, analyzing, and synthesizing evidence from a wide range of eligible studies. By following rigorous methodologies, comprehensive and critically appraised findings are produced, leading to evidence-based conclusions [127]. Despite their strengths, certain limitations may arise. More precisely, careful consideration should be given to the methodological quality of the eligible studies, as the study design parameters—sample size calculation, sample preparation, presence of control groups, observational conditions, and calibration of devices—have a considerable impact on the outcome of the study and the reliability of its findings [128]. Therefore, controversial results in in vitro studies should not be attributed solely to material-dependent factors but also to methodology-dependent variables and testing conditions. A typical example of inconsistency among in vitro studies refers to findings related to wear resistance, flexural strength, and elastic modulus. For instance, Degirmenci et al. and Rajabi et al. reported higher elastic modulus and flexural strength values of highly filled flowable composite resins compared to conventional composite resins [57,66]. Furthermore, Rajabi et al. and Checchi et al. showed a lower wear volume loss for highly filled flowable composite resins [64,66]. In contrast, Turk et al. and Imai et al. reported opposite findings, underscoring the variability in outcomes across studies [17,61]. Interestingly, Basheer et al. demonstrated similar flexural strength values among highly filled flowable composite resins and conventional composites but a higher elastic modulus for conventional composite materials [65]. Focusing on flexural strength, notable differences in specimen preparation across studies include variations in the polymerization process (e.g., light-curing duration and differences in the specific surfaces subjected to polymerization), storage process (e.g., storage in artificial saliva, in dark and dry environments, or in distilled water), and testing conditions (e.g., the presence or absence of thermocycling or prior immersion in various liquids). Regarding observations on wear resistance, discrepancies among studies may be attributed to several methodology-related factors such as differences in sample preparation (cylindrical vs. rectangular specimen shapes), sample conditioning (dry storage, storage in distilled water, or incubation for 24 h at 37 °C), variations in chewing simulation parameters ranging from 50.000 to 240.000 chewing cycles, differences in antagonist simulators (stainless steel balls, magnesium silicate-based balls, steatite balls), variations in the radius of the antagonist (ranging from 2.4 mm to 6 mm in diameter), and differences in the devices used for the assessment of wear (optical surface laser scanner, confocal laser scanning microscope, digital scanner without pre-wear baseline measurements). When newly introduced stand-alone restorative materials need to be evaluated, conventional nanofilled or nanohybrid composite resins—rather than traditional flowable composites—should serve as controls. Given that laboratory studies cannot simulate the complex oral environment to a great extent, in vitro studies are placed at the lowest level of the evidence-based medicine hierarchy [129]. Dental materials should perform optimally in the complex and variable oral environment, where masticatory forces, occlusal and dietary habits, temperature fluctuations, the formation of biofilms, enzyme activity, and salivary flow are constantly present. The interaction of these factors with dental materials may alter their physical, mechanical, and optical properties. Therefore, randomized controlled clinical trials provide stronger evidence regarding materials’ performance. Unfortunately, the number of eligible randomized controlled clinical trials is predictably low, and the trials are not readily comparable, since different restorative procedures were applied. For instance, one clinical trial investigated the clinical performance of highly filled flowable composite resins in cervical non-carious lesions [79], while others evaluated their clinical performance in class I and II cavities, in mid-size and extensive posterior restorations, ranging from occlusal caries to overlay replacement [78,80,81]. A high risk of bias arising from the randomization and allocation process is present in the clinical trial of Kitasako et al. in 2016 [81], where dice were used for randomization, and allocation concealment was not ensured. In contrast, the other three clinical trials used automated random sequence generators to minimize randomization bias. Additionally, the same clinical trial lacked a sample size calculation, resulting in a low statistical power. All four RCTs had limited sample sizes. Hançer Sarıca et al. in 2025 included 259 class II restorations at baseline [84], whereas a relatively smaller amount of participants was present in the other three clinical trials. Dropouts further reduced the number of restorations evaluated after baseline. Despite these drawbacks, no bias regarding the outcome measurement was detected, as acceptable evaluation criteria (FDI criteria and modified USPHS criteria) were employed. Limitations involving randomization, sample size, dropout rates, and short term follow-up periods must be taken into consideration before drawing definite conclusions [130].

It should not be overlooked that the clinical performance of composite resin materials in the oral environment is influenced by multiple factors, including cavity size, tooth position, the restorative techniques applied (rubber dam isolation, the polishing systems employed), the patient’s profile (e.g., caries risk, the presence of parafunctional habits), and the operator’s skills. By minimizing the risk factors, clinical failures such as fractures, secondary caries, and esthetic complications can be significantly reduced [131].

Emphasis should be given to the establishment of well-designed clinical trials in order to unfold the full potential of these novel restorative materials. Additionally, it would have been valuable to assess the behavior of highly filled flowable resin-based materials in oral conditions by conducting in situ studies, which may reveal a potential connection between optical parameters, mechanical properties, surface characteristics, saliva, and the oral microbiome.

## 5. Conclusions

Highly filled flowable resin composites display excellent optical properties in laboratory studies, supporting their clinical use as esthetic restorative materials in the anterior region. Based on the results of this systematic review, they also appear to be suitable for minimally invasive restorations in the posterior region. Given the limited number of randomized controlled trials and the notable limitations of the included in vitro studies, the clinical application of these materials in load-bearing areas and in patients with significant tooth wear or heavy parafunctional activity should be avoided.

## Figures and Tables

**Table 1:** Database search strategy and identification of studies.

Database	Search Terms	Results
PubMed	✓ “highly filled flowable composite resin” ✓ ((highly filled flowable composite resin) OR (injectable composite resin) OR (newly developed flowable resin)) AND ((mechanical properties) OR (physical properties) OR (wear resistance) OR (surface roughness) OR (hardness) OR (flexural strength) OR (optical properties) OR (color)) ✓ ((highly filled flowable composite resin) OR (injectable composite resin) OR (newly developed flowable resin)) AND ((clinical trials) OR (in vitro studies))	295
Cochrane Library	✓ “highly filled flowable composite resin”	39
Scopus	✓ highly AND filled AND flowable AND composite AND resin ✓ ((highly AND filled AND flowable AND composite AND resin) OR (injectable AND composite AND resin) OR (newly AND developed AND flowable AND resin)) AND ((clinical AND trials) OR (in AND vitro AND studies)) ✓ ((highly AND filled AND flowable AND composite AND resin) OR (injectable AND composite AND resin) OR (newly AND developed AND flowable AND resin)) AND ((mechanical AND properties) OR (physical AND properties) OR (wear AND resistance) OR (surface AND roughness) OR (hardness) OR (flexural AND strength) OR (optical AND properties) OR (color))	182
Manual search	✓ Reference listing	6
	Total studies identified	522

**Table 2:** Summary of eligible studies based on research type and investigated parameters.

Author/Year	Research Type	Investigated Outcomes
**15 in vitro studies incorporating conventional composite resins into their methodological framework**		
Nair et al., 2017 [55]	in vitro	color stability/microhardness
Imai et al., 2019 [17]	in vitro	wear volume loss/maximum depth of loss/flexural strength/flexural modulus/thread formation/inorganic filler content
Korkut et al., 2020 [56]	in vitro	color stability
Degirmenci et al., 2022 [57]	in vitro	surface roughness/microhardness/flexural strength/elastic modulus
Uctasli et al., 2023 [58]	in vitro	color stability
Islam et al., 2023 [59]	in vitro	microhardness/water sorption/water solubility/color stability
Degirmenci et al., 2023 [60]	in vitro	translucency/opalescence/chroma/surface roughness
Turk et al., 2024 [61]	in vitro	wear volume loss/maximum depth of loss
Tüter Bayraktar et al., 2024 [62]	in vitro	fluorescence adjustment level/color adjustment level
Gerges et al., 2024 [63]	in vitro	fracture resistance/mode of failure
Checchi et al., 2024 [64]	in vitro	surface roughness/wear resistance
Basheer et al., 2024 [65]	in vitro	flexural strength/elastic modulus/surface roughness/microhardness/microleakage
Rajabi et al., 2024 [66]	in vitro	flexural strength/wear resistance (wear volume loss)
Francois et al., 2024 [67]	in vitro	flexural strength/wear resistance
Jrady et al., 2024 [68]	in vitro	color stability
**12 in vitro studies without the incorporation of conventional composite resins in their methodology**		
Lai et al., 2018 [69]	in vitro	surface roughness/surface gloss/color stability
Ujiie et al., 2020 [70]	in vitro	wear resistance (wear volume loss—maximum depth of wear)
Shimatani et al., 2020 [71]	in vitro	cuspal deflection/flexural strength/elastic modulus
Tsujimoto et al., 2021 [72]	in vitro	flexural strength/elastic modulus/shear bond strength/marginal adaptation/polymerization shrinkage/polymerization shrinkage stress
Ludovichetti et al., 2022 [73]	in vitro	microhardness/surface roughness/depth of cure (DOC)/filler dimension
Elsahn et al., 2023 [74]	in vitro	microhardness/surface roughness/wear volume loss
Elgammal et al., 2023 [75]	in vitro	surface roughness/surface gloss
Vulović et al., 2023 [76]	in vitro	surface roughness/microbial adhesion/cell viability
Chen et al., 2024 [77]	in vitro	surface roughness/wear volume loss/maximum depth of wear/microbial adhesion/cell viability/biocompatibility
Bai et al., 2024 [78]	in vitro	water sorption/water solubility/elemental release/degree of conversion/water contact angle/color stability
Miyashita-Kobayashi et al., 2024 [79]	in vitro	surface gloss/surface roughness/color stability
Vulović et al., 2024 [80]	in vitro	surface roughness/microhardness
**4 randomized controlled clinical trials with highly filled flowable composite resins**		
Kitasako et al., 2016 [81]	RCT	clinical performance of mid-size to extensive posterior restorations after 36 months.
Zhang et al., 2021 [82]	RCT	clinical performance of non-carious cervical lesions (NCCLs) after 3 years
Elderiny et al., 2024 [83]	RCT	clinical performance of Class I and II restorations after 18 months
Hançer Sarıca et al., 2025 [84]	RCT	clinical performance of Class II restorations after 2 years

**Table 3:** In vitro studies incorporating conventional composite resins into their methodological framework.

Author/Year	Dental Materials and Procedures	Tested Parameters and Key Findings
Nair et al., 2017 [55]	(1) G-aenial Universal Flo (GC Corp., Tokyo, Japan) (2) Filtek Z350XT (3M ESPE, St. Paul, MN, USA) (3) Tetric N Ceram (Ivoclar Vivadent AG, Schaan, Lichtenstein) immersion in coffee + tooth brushing simulation	Color stability + microhardness: Inferior optical and mechanical properties of highly filled flowable composite resin
Imai et al., 2019 [17]	Experimental groups: Six flowable composite resins: (1) Beautifil Flow Plus F00 (BF; Shofu Inc., Kyoto, Japan) (2) Clearfil Majesty ES Flow (CE; Kuraray Noritake Dental Inc., Tokyo, Japan) (3) Estelite Universal Flow (EU; Tokuyama Dental Corp, Tokyo, Japan) (4) Filtek Supreme Ultra Flowable Restorative (FS; 3M ESPE, St. Paul, MN, USA) (5) G-ænial Universal Flo (GU) (6) Gracefil Zero Flow (GZ; GC Corp., Tokyo, Japan) Control groups: Two conventional composite resins: (1) Microhybrid, Clearfil AP-X (AP; Kuraray Noritake Dental Inc., Tokyo, Japan) (2) Nanofilled composite resin, Filtek Supreme Ultra (SU; 3M ESPE, St. Paul, MN, USA) Polishing procedure: Grinding up to 1200-grit by silicon carbide paper discs (SiC paper discs) + wear simulation by the use of stainless steel balls as antagonists (50,000 cycles)	Wear volume loss/maximum depth of loss/flexural strength/flexural modulus/thread formation/inorganic filler content: Highly filled flowable resins present significantly lower inorganic filler content elastic modulus values significantly higher resilience thread formation volume loss compared to conventional composite resins Increasing the inorganic filler content did not enhance the physical properties of highly filled flowable composite resins
Korkut et al., 2020 [56]	(1) Two high-viscosity highly filled flowable composite resins (G-aenial Universal Injectable, GC, Tokyo, Japan; Estelite Super Low Flow, Tokuyama Dental, Tokyo, Japan) (2) A bulk-fill flowable composite resin (Filtek Bulk-Fill Flowable) (3) A low-viscosity flowable composite resin (Filtek Ultimate Flowable, 3M ESPE, St. Paul, MN, USA) (4) A conventional composite resin (Filtek Ultimate, 3M ESPE, St. Paul, MN, USA) Polishing procedure: Sof-Lex polishing discs (3M ESPE, St. Paul, MN, USA) Experimental groups: Immersion in various colorant solutions Control group: Immersion in saline	Color stability: Traditional flowable composite resins presented the highest level of color change in all time intervals Highly filled flowable composite resins presented a comparable color stability to conventional composites
Degirmenci et al., 2022 [57]	(1) Highly filled flowable composite resin (G-aenial Universal Injectable, GC) (2) Bulk-fill flowable composite resin (Estelite Bulk Fill Flow, Tokuyama Corp., Tokyo, Japan) (3) Conventional microhybrid composite resin (G-aenial Posterior, GC Europe N.V., Leuven, Belgium) Polishing procedure: Grinding up to 1200-grit by SiC paper discs + ultrasonication + immersion into Coke Orange juice Artificial saliva (control group) measured Before exposure to beverages On the first day First week First month First year no thermocycling	Microhardness values: Microhybrid > bulk-fill > highly filled flowable Elastic modulus: Bulk-fill > highly filled flowable > microhybrid composite Flexural strength: Highly filled flowable > bulk-fill > microhybrid composite Surface roughness: Highly filled flowable > bulk-fill > microhybrid composite The highly filled flowable composite resin exhibited acceptable flexural strength values
Uctasli et al., 2023 [58]	(1) Filtek Universal Restorative (3M ESPE) (2) SDR flow + (Dentsply Sirona, York, PA, USA) (3) everX Flow (GC Corp, Tokyo, Japan) (4) G-ænial A’CHORD (GC Corp, Tokyo, Japan) (5) G-ænial Universal Flo (GC) (6) G-ænial Universal Injectable (GC) Polishing procedure: By machine (1000-grit, 2000-grit, and 4000-grit SiC paper discs) By hand (3 M Sof-Lex Polishing System) + immersion in different beverages: Distilled water Coffee Red wine Energy drink Coke	Color stability: The flowable composites (traditional and highly filled) showed similar ΔΕ values to the conventional composite materials in the hand-polished groups. Repolishing serves as an effective technique for eliminating surface discoloration in composite restorations
Islam et al., 2023 [59]	(1) Beautifil Injectable X (highly filled flowable resin-based material), Shofu, Kyoto, Japan (2) Beautifil II LS (low-shrinkage paste resin-based material), Shofu, Kyoto, Japan (3) CharmFil flow (flowable composite resin), Dentkist Inc, Gunpo-si, Gyeonggi-do, Korea (4) CharmFil Plus (nanofilled composite resin), Dentkist Inc, Gunpo-si, Gyeonggi-do, Korea + thermocycling and staining challenge	Microhardness: Conventional composite resin > highly filled flowable > traditional flowables Water sorption: Beautifil Injectable X and II LS showed a negative WS Color stability: All groups showed significant color alterations after one week of staining challenge
Degirmenci et al., 2023 [60]	(1) Microhybrid conventional composite resin (G-ænial Anterior, GC, Tokyo, Japan) (2) Highly filled flowable composite resin (G-ænial Universal Flo) (3) Highly filled flowable composite resin (G-ænial Universal Injectable) Polishing procedure: 1200-grit SiC paper discs Experimental groups: Multi-step rubber polishing discs (Sof-Lex polishing discs, 3M ESPE, St. Paul, MN, USA) Two-step flexible polishing discs (CLEARFIL Twist DIA, EVE Ernst Vetter, Keltern, Germany) Control group: no commercial polishing	Translucency/opalescence/chroma: G-aenial Universal Injectable had the highest translucency and opalescence and the lowest chroma value Polishing procedure did not significantly affect the refractive index Surface roughness: Composite type and polishing procedure show statistical significant effects on surface roughness
Turk et al., 2024 [61]	Experimental groups: (1) Nanohybrid conventional (G-aenial Posterior) and flowable (G-aenial Universal Injectable) composite resins (2) Nanofilled bulk (Filtek One Bulk-fill Restorative) and flowable (Filtek Ultimate Flow) composite resins (3) Submicron-filled conventional (Estelite Posterior Quick, Tokuyama, Tokyo, Japan) and flowable (Estelite Bulk-Fill Flow, Tokuyama, Tokyo, Japan) composite resins Control group: Buccal surfaces of extracted human premolars (4) + thermomechanical chewing simulation for 240,000 cycles by the use of magnesium silicate-based balls as antagonists	Wear volume loss and loss depth: Nanofilled > nanohybrid = submicron-filled composite resins Flowable composites > conventional composites Highly filled flowable composite resins still display inferior wear resistance compared to conventional composite resins.
Tüter Bayraktar et al., 2024 [62]	Class V cavities restored by five paste-type resin composites: (1) Omnichroma (Tokuyama Dental Corporation, Tokyo, Japan) (2) G-aenial A-Chord (3) Estelite Asteria (Tokuyama Dental Corporation, Tokyo, Japan) (4) Clearfil Majesty ES-2 (Kuraray Noritake, Tokyo, Japan) (5) Charisma Diamond One (Kulzer Dental, Hanau, Germany) and five highly-filled flowable composites: (6) Omnichroma flow (Tokuyama Dental Corporation, Tokyo, Japan) (7) G-aenial Universal Injectable (GC) (8) Estelite Universal Flow (Tokuyama Dental Corporation, Tokyo, Japan) (9) Clearfil Majesty Low Flow (Kuraray Noritake, Tokyo, Japan) (10) Charisma Diamond One Flow (Kulzer Dental, Hanau, Germany) Polishing procedure: two-step diamond spiral wheels	Fluorescence adjustment level/color adjustment level: Paste-type composites presented significantly lower ΔE_FI_ and ΔE_CP_ values than the highly filled flowable composites The only clinically acceptable color adjustment was found for G-aenial Universal Injectable among the flowable composites.
Gerges et al., 2024 [63]	50 extracted maxillary premolars Control group: 10 intact, untreated premolars Experimental groups (40 extracted teeth) Small class II cavities restored with highly filled flowable resin (G-aenial Universal Injectable, GC) Extensive class II cavities restored with highly filled flowable resin Small class II cavities restored with conventional composite resin (G-aenial Posterior) Extensive class II cavities restored with conventional composite resin	Fracture resistance/mode of failure: no statistically significant differences between the fracture resistance the mode of failure in all five groups
Checchi et al., 2024 [64]	Experimental groups: Two highly filled flowable composites: (1) Clearfl Majesty ES flow (Kuraray Noritake) (2) G-aenial Universal Injectable (GC) Control groups: Two conventional resin composites: (3) Clearfl Majesty ES-2 (Kuraray Noritake) (4) G-aenial A’CHORD (GC) Polishing procedure: Up to 4000-grit SiC paper for 20 s + chewing simulation by the use of a steatite sphere as antagonist (240.000 cycles, 20N)	Surface roughness/wear resistance: Surface roughness and wear of highly filled flowable composites were comparable to that of conventional composites Highly filled flowables can be used in occlusal areas, especially when overcured
Basheer et al., 2024 [65]	Experimental groups: Four highly filled flowable composites: (1) G-aenial Universal Flo (2) G-aenial Universal Injectable (3) Beautifl Injectable X (4) Beautifl Flow Plus Control group: Nanohybrid conventional resin composite (5) Filtek Z350 XT, 3M ESPE thermocycling prior to investigation	Flexural strength: no statistically significant difference between all highly filled flowables and the control Elastic modulus: Filtek Z350 presented a higher elastic modulus compared to experimental groups Microhardness (VHN): Conventional composite > highly filled flowable composite Surface roughness: No differences between groups Microleakage: Conventional composite resin > highly filled flowables
Rajabi et al., 2024 [66]	(1) G-aenial Universal Injectable (2) Beautifil Plus F00 (3) Tetric EvoFlow and a conventional composite resin (control group): (4) Empress Direct (Ivoclar Vivadent AG, Schaan, Lichtenstein) + wear simulation (200,000 cycles) by the use of steatite balls as antagonists no thermocycling prior to investigation	Flexural strength/wear resistance (wear volume loss): G-aenial Universal Injectable and Beautifil Plus F00 presented statistically lower volume loss statistically higher mean flexural strength values compared to conventional composite and Tetric EvoFlow Highly filled flowable composite resins may be suitable to use in occlusal, load-bearing areas
Francois et al., 2024 [67]	Nine highly filled flowable resin composites + viscous composites + traditional flowable composites	Flexural strength/wear resistance: Most highly filled composites exhibited - similar flexural strength - superior wear resistance compared to viscous composites. Elastic modulus: Conventional composite resins > highly filled flowables > traditional flowables
Jrady et al., 2024 [68]	(1) Microhybrid composite resin (Gradia Direct Anterior, GC Corp., Tokyo, Japan) (2) Nanohybrid composite resin (Palfique LX5, Tokuyama Dental Corp, Tokyo, Japan) (3) Nanofilled composite resin (Filtek Universal, 3M ESPE, St. Paul, MN, USA) (4) Highly filled flowable composite resin (G-aenial Universal Injectable, GC) Polishing procedures: (1) No polishing (control group) (2) 4-step polishing using aluminum oxide discs (Sof-Lex polishing discs, 3M ESPE) (3) 3-step polishing using silicon rubber diamond discs (Astropol, Ivoclar Vivadent, Schaan, Lichtenstein) (4) 1-step polishing (Charisma Easyshine, Kulzer Dental, Hanau, Germany) + immersion in water or coffee	Color stability: Material type, polishing technique, storage media, and their interaction influence ΔΕ values. The lowest color change is present in Highly filled flowable composite resin compared to other materials 3-step polishing diamond discs compared to other polishing procedures Water compared to coffee Highly filled flowable composite resin is more resistant to staining

**Table 4:** In vitro studies conducted without the incorporation of conventional composite resins in their methodology.

Author/Year	Dental Materials and Procedures	Tested Parameters and Key Findings
Lai et al., 2018 [69]	(1) Four traditional flowable composites: GrandioSO Flow (VOCO GmbH, Cuxhaven, Germany), Arabesk Flow (VOCO GmbH, Cuxhaven, Germany) Kerr Revolution Formula 2 (Kerr, Orange, CA, USA) Gradia Direct LoFlo (GC Corporation, Tokyo, Japan) (2) One self-adhering flowable composite: Kerr Vertise Flow (Kerr, Orange, CA, USA) (3) One highly filled flowable composite: G-ænial Universal Flo (GC) Experimental groups: Toothbrushing simulation Control groups: No toothbrushing simulation Polishing procedure: Grinding up to 4000-grit SiC papers + ultrasonication	Surface roughness/surface gloss/color stability: Highly filled flowable composite showed the highest gloss value the lowest Ra values (0.11 μm) Color alteration of all composites was acceptable
Ujiie et al., 2020 [70]	(1) Filtek Bulk Fill Flowable Restorative (3M ESPE, St. Paul, MN, USA) (2) G-aenial Bulk Injectable (GC Corp., Tokyo, Japan) (3) SDR flow + (Dentsply, York, PA, USA) (4) Tetric EvoFlow Bulk Fill (Ivoclar Vivadent AG, Schaan, Lichtenstein) (5) Clearfil Majesty IC (Kuraray Noritake Dental Inc., Tokyo, Japan) (6) Filtek Supreme Ultra Flow (3M ESPE) (7) G-aenial Universal Flo (GC) (8) Herculite XRV Ultra Flow (Kerr, Orange, CA, USA) Universal polishing procedure: Grinding up to 4000-grit by SiC paper discs + wear simulation by 400,000 cycles	Wear resistance (wear volume loss—maximum depth of wear): Highly filled flowable composites (G-aenial bullk injectable, G-aenial Universal Flo and Filtek Supreme Ultra Flow) showed significantly less wear and significantly lower volume loss than the other flowable materials
Shimatani et al., 2020 [71]	Five bulk-fill flowable composite resins: (1) Beautifil Bulk Flowable (BF; Shofu, Kyoto, Japan) (2) Bulk Base (BB; Sun Medical, Shiga, Japan) (3) Filtek Fill and Core (FF; 3M ESPE, St Paul, MN, USA) (4) SDR (SD; Dentsply Sirona, York, PA, USA) (5) X-tra base (XB; VOCO GmbH, Cuxhaven, Germany) and six conventional flowable resin composites: (6) Clearfil Majesty ES Flow, Kuraray Noritake (CE) (7) Clearfil Majesty LV, Kuraray Noritake (CL) (8) Estelite Universal Flow, Tokuyama Dental Corp. (EU) (9) G-ænial Universal Injectable, GC (GI) (10) Filtek Supreme Ultra Flowable, 3M ESPE (FS) (11) UniFil LoFlo Plus (UF; GC, Tokyo, Japan). Polishing procedure: SiC papers of 600—grit size.	Cuspal deflection: conventional flowable resin composites > bulk fill flowable resin composites Flexural strength and elastic modulus: Highly filled flowable resin composites > bulk fill flowable resin composites
Tsujimoto et al., 2021 [72]	4 highly filled flowable composites: (1) Beautifil Flow Plus X F03 (BF) (2) Clearfil Majesty ES Flow Low (CM) (3) Estelite Universal Flow Medium Flow (EU) (4) G-ænial Universal Injectable (GU). Traditional flowable composite (5) Unifil LoFlow Plus (UP, GC, Tokyo, Japan) Bulk-fill flowable composite (6) Filtek Bulk Fill Flowable (FF) Two time intervals for polymerization shrinkafe t1: 10 min after curing t2: 24 h after curing	Flexural strength/elastic modulus/shear bond strength/marginal adaptation/polymerization shrinkage/polymerization shrinkage stress: Highly filled flowable composites showed significantly higher flexural strength values immediately after and 24 h after polymerization elastic modulus after 24 h polymerization shrinkage stress compared to traditional and bulk-fill flowable composites Highly filled flowable composites showed a similar bond strength marginal adaptation compared to traditional and bulk-fill composites regardless of the storage conditions
Ludovichetti et al., 2022 [73]	Bulk-fill flowable composite resins: (1) Filtek Bulk Fill Flowable Restorative (bulk fill flowable), (2) Tetric EvoFlow Bulk Fill (bulk fill flowable) Highly filled flowable composite resin: (3) G-ænial Universal Injectable (highly filled flowable composite) Traditional flowable composite resin: (4) G-ænial Flo X (microhybrid flowable composite) (5) Filtek Supreme XTE Flowable Restorative (nanofilled flowable)	DOC: Bulk-fill flowables > highly filled flowable > traditional flowables Microhardness: Bulk fill flowables = highly filled injectable > traditional flowable resin Surface roughness: Bulk fill flowables > highly filled flowables
Elsahn et al., 2023 [74]	1 mm thin, conservative occlusal veneers fabricated by (1) Cerasmart blocks, Cerasmart, GC, Tokyo, Japan (CS) (indirect CAD/CAM technique) (2) Beautifil Injectable X (BF) (3) G-ænial Universal Injectable (GU) (4) SonicFill 2, Kerr, Orange, CA, USA (SF) Polishing procedure: Two-step composite finishing and polishing set + thermomechanical cyclic loading	Microhardness: CS> GU =SF > BF Surface roughness: SF > BF > CS > GU Volumetric wear: SF > BF > CS > GU GU injectable occlusal veneers are less influenced by thermomechanical cyclic loading than CS milled veneers BF and SF: significant volumetric loss and increased Ra values
Elgammal et al., 2023 [75]	G-aenial Bulk Injectable, GC (highly filled, bulk flowable composite resin) Polishing procedures: Two-step Sof-Lex spiral wheels system (3M ESPE, St. Paul, MN, USA) Multiple-step Sof-Lex discs (3M ESPE) 2 different time intervals: After polishing After three months immersed in 2 different liquids: (a) Artificial saliva (control group) (b) Coca-Cola	Surface roughness/surface gloss: Improved surface roughness and gloss by using the multiple-step polishing system Acidic media had a negative impact on surface roughness and surface gloss of the resin composite material
Vulović et al., 2023 [76]	(1) Filtek Supreme Flowable Restorative, 3M ESPE (FF) (2) Tetric EvoFlow, Ivoclar Vivadent (TEF) (3) G-aenial Universal Flo, GC (GUF) (4) G-aenial Universal Injectable, GC (GUI) Polishing procedures: Sof-Lex discs, 3M ESPE, St. Paul, MN, USA (SLD) Sof-Lex Spirals, 3M ESPE, St. Paul, MN, USA (SLS) One Gloss, Shofu, Tokyo, Japan (OG) PoGo, Dentsply/Caulk, Milford, DE, USA (PG)	Surface roughness/microbial adhesion/cell viability: Both material and polishing procedures affect surface roughness and microbial adhesion GUI adhered the lowest amount of *Strep.mutans*, due to the smoothest surfaces The smoothest surfaces possess GUI and GUF, among materials, and SLD and SLS, among polishing procedures
Chen et al., 2024 [77]	Three highly filled flowable composites: (1) G-aenial Universal Injectable, (GU) (2) Beautifil Injectable XSL (BI), (3) Filtek Supreme Flowable (FS) and a compomer: (4) Dyract Flow, Dentsply, York, PA, USA (DF) Polishing procedure: SiC abrasive papers (600-, 1000-, 2000-grit size) time intervals of investigation: Immediately after preparation After 1 day 7-day 14-day 30-day water storage	Surface roughness/wear volume loss/maximum depth of wear/microbial adhesion/cell viability/biocompatibility: Mechanical properties are material-dependent and sensitive to water storage CFU counting: No significant differences between the materials GU and FS had a more favorable cell adhesion and morphology FS presented a slightly thicker biofilm, and BI showed a lower bacterial density Flexural strength: GU > FS > BI > DF at all testing levels Superior properties of highly filled injectable composite resins compared to compomers
Bai et al., 2024 [78]	Highly filled flowable resin composites + compomer (1) G-aenial Universal Injectable (GU) (2) Beautifil Injectable XSL (BI), (3) Filtek Supreme Flowable (FS) (4) Dyract Flow (DF) Polishing procedure: SiC apapers (up to 2000-grit)	Chemical properties/color stability: G-aenial Universal Injectable exhibits favorable water sorption values: GU < FS < BI < DF favorable water solubility values: GU = BI < FS < DF) FS presents the lowest elemental release the best color stability the highest degree of conversion GU and BI had the largest water contact angle Both material type and duration of water storage affected the optical properties
Miyashita-Kobayashi et al., 2024 [79]	Estelite Universal Flow (EUF) Beautifil Flow Plus F00 (BFP) GC Fuji II, GC, Tokyo, Japan (FLC) GC Fuji IX GP EXTRA, GC, Tokyo, Japan (FGP) Prophylaxis procedures: Group 1: Load of 100 gf, 10 s, 4× Group 2: Load of 100 gf, 30 s, 4× Group 3: Load of 300 gf, 10 s, 4× Group 4: Load of 300 gf, 30 s, 4×	Surface roughness/surface gloss/color stability: Highly filled flowable resins presented favorable surface characteristics compared to glass ionomer cements
Vulović et al., 2024 [80]	Four flowable composite resins: (1) G-aenial Universal Flo (2) G-aenial Universal Injectable (3) Tetric EvoFlow (4) Filtek Supreme Flowable Restorative before immersion, 9 h and 18 h after immersion in different media: gastric juice, fizzy drink, citric juice, and artificial saliva (control group)	Surface roughness/microhardness: G-aenial Universal Injectable exhibited a lower surface roughness and higher hardness compared to other highly filled flowable composite resins both before and after exposure to acidic media

**Table 5:** Randomized controlled clinical trials with highly filled flowable composite resins.

Author/Year	Objective	Materials	Sample Size/Time Intervals	Evaluation Criteria	Results
Kitasako et al., 2016 [81]	Mid-size to extensive posterior restorations after 36 months.	1. Conventional composite resin (Estelite Sigma Quick, Tokuyama, Tokyo, Japan) 2. Highly filled flowable composite resin (G-aenial Universal Flo, GC) two-step self-etch adhesive applied to both materials	58 mid-size to extensive posterior composite restorations in 32 patients Restoration evaluation: a. After placement b. 6 months c. 12 months d. 24 months e. 36 months After 36 months 42 restorations were evaluated in 21 patients	World Dental Federation (FDI) criteria	No statistically significant difference between cavities restored with highly filled flowable and conventional composite resins No secondary caries observed.
Zhang et al., 2021 [82]	Non-carious cervical lesions (NCCLs) after 3 years	1. Highly filled flowable composite (Clearfil Majesty ES Flow, Kuraray Noritake Dental Inc., Tokyo, Japan) 2. Conventional paste-type composite (Clearfil Majesty ES-2, Kuraray Noritake Dental Inc., Tokyo, Japan) Clearfil SE Bond (Kuraray Noritake Dental Inc., Tokyo, Japan)	84 NCCLs in 27 subjects were included Restoration evaluation: a. baseline (BL) b. 1 year c. 2 years d. And 3 years	FDI criteria	No significant difference between the two material groups at any time interval concerning functional properties The highly filled flowable resin composite presented a significantly better surface luster (*p* < 0.01) at the 1-year recall marginal staining (*p* < 0.05) at any time point
Elderiny et al., 2024 [83]	Class I and II restorations after 18 months	1. Bioactive highly filled flowable resin composite (Beautifil Flow Plus X F00, Shofu Inc., Kyoto, Japan) 2. Nanohybrid resin composite (Tetric N-Ceram, Ivoclar Vivadent AG, Schaan, Lichtenstein)	18 patients with 26 class I and II carious cavities Restoration evaluation: a. Baseline b. 6 months c. 12 months d. 18 months	modified United States Public Health Service (USPHS) criteria	No statistically significant difference between materials at different time intervals in terms of anatomical form, secondary caries, marginal staining, postoperative sensitivity (*p* = 0.99), and marginal adaptation (*p* > 0.05)
Hançer Sarıca et al., 2025 [84]	Class II restorations after 2 years	1. Conventional composite: Clearfil Majesty Posterior (Kuraray Noritake Dental Inc., Tokyo, Japan) 2. Bulk-fill composite: Filtek One Bulk Fill Restorative (Solventum) 3. Highly filled flowable composite: G-aenial Universal Injectable (GC, Japan)	110 patients with 259 class II restorations evaluated: a. At baseline b. After 1 year c. After 2 years After 2 years: A. 59 conventional composite restorations B. 68 bulk-fill composite restorations C. 61 highly filled flowable composite restorations in 74 patients have been evaluated	FDI criteria	The highly filled flowable composite and the bulk-fill composite presented a better clinical performance regarding surface gloss compared to the conventional composite (*p* < 0.05)

## Data Availability

No new data were created or analyzed in this study. Data sharing is not applicable to this article.
